# *Echinacea purpurea* Polysaccharide Ameliorates Dextran Sulfate Sodium-Induced Colitis by Restoring the Intestinal Microbiota and Inhibiting the TLR4-NF-κB Axis

**DOI:** 10.3390/nu16091305

**Published:** 2024-04-26

**Authors:** Fan-Hao Wei, Wen-Yin Xie, Pei-Sen Zhao, Wei Gao, Fei Gao

**Affiliations:** Department of Laboratory Animals, College of Animal Sciences, Jilin University, Changchun 130062, China; weifh22@mails.jlu.edu.cn (F.-H.W.); wyxie22@mails.jlu.edu.cn (W.-Y.X.); zhaops22@mails.jlu.edu.cn (P.-S.Z.); gaowei81@jlu.edu.cn (W.G.)

**Keywords:** *Echinacea purpurea* polysaccharide (EPP), ulcerative colitis (UC), inflammation, intestinal microbiota, TLR4/NF-κB, intestinal barrier

## Abstract

Ulcerative colitis (UC) is a chronic intestinal ailment which cannot be completely cured. The occurrence of UC has been on the rise in recent years, which is highly detrimental to patients. The effectiveness of conventional drug treatment is limited. The long-term usage of these agents can lead to substantial adverse effects. Therefore, the development of a safe and efficient dietary supplement is important for the prevention of UC. *Echinacea purpurea* polysaccharide (EPP) is one of the main bioactive substances in *Echinacea purpurea*. EPP has many favorable effects, such as antioxidative, anti-inflammatory, and antitumor effects. However, whether EPP can prevent or alleviate UC is still unclear. This study aims to analyze the effect and mechanism of EPP on UC in mice using a 3% dextran sulfate sodium (DSS)-induced UC model. The results showed that dietary supplementation with 200 mg/kg EPP significantly alleviated the shortening of colon length, weight loss, and histopathological damage in DSS-induced colitis mice. Mechanistically, EPP significantly inhibits the activation of the TLR4/NF-κB pathway and preserves the intestinal mechanical barrier integrity by enhancing the expression of claudin-1, ZO-1, and occludin and reducing the loss of goblet cells. Additionally, 16S rRNA sequencing revealed that EPP intervention reduced the abundance of Bacteroides, Escherichia–Shigella, and Klebsiella; the abundance of Lactobacillus increased. The results of nontargeted metabonomics showed that EPP reshaped metabolism. In this study, we clarified the effect of EPP on UC, revealed the potential function of EPP, and supported the use of polysaccharide dietary supplements for UC prevention.

## 1. Introduction

Ulcerative colitis (UC) is a chronic inflammatory disease that affects the colon, is characterized by recurrent attacks, and cannot be cured [1]. The occurrence of UC is growing annually, and UC has become a worldwide public health concern [2]. The pathogenesis of UC can be driven by hereditary diseases; unhealthy lifestyles, such as staying up late and smoking; psychological problems, such as depression; and intestinal barrier and immune factors. 5-Aminosalicylate (5-ASA) and steroids are usually used for the treatment of mild-to-moderate UC but have several limitations. Biological drugs such as immunosuppressants have serious side effects. Therefore, providing ulcerative colitis patients with targeted treatment that has less toxicity and fewer side effects is necessary and can improve the quality of life.

*Echinacea purpurea* is a botanical drug with a long history of use in Europe and North America for the treatment of upper respiratory tract infections [3]. Echinacea has a variety of bioactive components, including flavonoids, polysaccharides, glycoproteins, caffeic acid derivatives, and others [4,5]. The pharmacological activity of Echinacea also depends on its bioactive components (such as anti-inflammatory agents, immune regulation agents, and bacteriostasis agents) [6,7,8]. *Echinacea purpurea* polysaccharide has strong anti-inflammatory properties. *Echinacea purpurea* polysaccharide can significantly reduce the levels of inflammatory factors such as TNF-α and IL-6 in an LPS-induced septicemia model and upregulate the expression of the anti-inflammatory factor IL-10 to alleviate the inflammatory response induced by LPS [9]. Moreover, *Echinacea purpurea* polysaccharide downregulates the expression of TNF-α, IL-1β, and IL-6 induced by LPS; inhibits LPS-induced apoptosis; and plays a role in the TLR4/NF-κB signaling pathway. In addition, *Echinacea purpurea* polysaccharide can reduce oxidative stress in vitro and in vivo, and its antioxidant ability has been confirmed by free radical scavenging experiments; furthermore, *Echinacea purpurea* polysaccharide participates in mitochondrial apoptosis and the Keap1-Nrf2-ARE signaling pathway [10]. However, whether active polysaccharides from *Echinacea purpurea* can prevent colitis remains unknown.

Dextran sodium sulfate (DSS) has been widely used to establish experimental animal models of UC [11]. The clinical phenotype of the UC models is highly similar to that of patients with ulcerative colitis [12], which includes the upregulation of inflammatory factor expression [13], intestinal barrier damages, increased intestinal permeability [11], the destruction of the intestinal microbial balance [14], and disorder in metabolism [15].

Our study aims to evaluate the preventative effects of EPP intervention on DSS-induced inflammation and oxidative damage and the impact of EPP on the destruction of intestinal machinery, intestinal microbiota, and their metabolites in the microbial barrier in a mouse model of acute colitis.

## 2. Materials and Methods

### 2.1. Materials

*Echinacea purpurea* polysaccharide was acquired from Shanxi Rongling Biotechnology Co. (Xi’an, China). ZO-1, occludin, and claudin-1 primary antibodies were acquired from Affinity Biosciences (Cincinnati, OH, USA). TLR4, GAPDH, β-actin, p-NF-κB-P65, and NF-κB-P65 primary antibodies were acquired from Cell Signaling Technology (2118, Shanghai, China).

### 2.2. Laboratory Animals

Thirty-six male BALB/c mice, 6 weeks old and weighing 20 ± 2 g, were procured from Liaoning Changsheng Biotechnology Company in Benxi, China. Approval for our research was granted by the Animal Ethics and Welfare Committee of Jilin University (license No. SY202306064).

### 2.3. Colitis Model and Treatment

**Figure 1:**
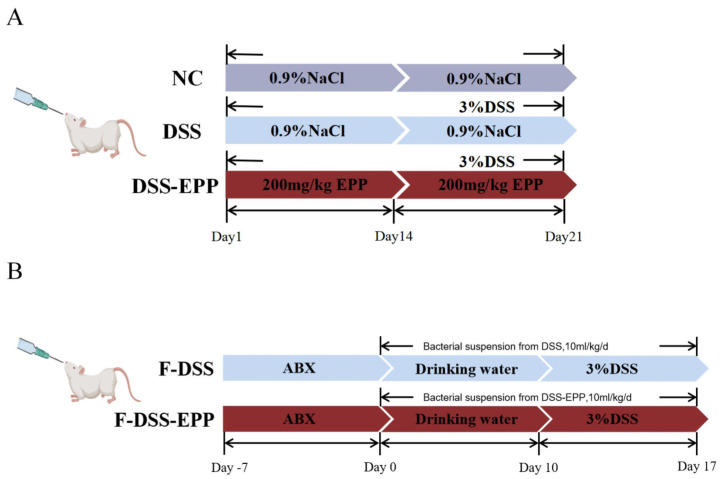
Experimental flow chart of animal treatments. (**A**) Flow chart of colitis model and EPP treatment. (**B**) Flow chart of fecal microbial transplantation.

The mice were raised in a barrier environment, and the formal experiment began after 7 days of adaptation. As shown in Figure 1A, a total of 24 mice were grouped as follows: the normal control group (NC), the DSS-induced colitis model group (DSS), and the *Echinacea purpurea* polysaccharide treatment group (DSS-EPP). Mice in the NC group were orally gavaged 0.9% NaCl saline for 21 days. The DSS-EPP group was orally administered EPP (200 mg/kg/d, dissolved in 0.9% NaCl) by gavage for 21 days (according to previous studies, the concentration of EPP used was at 50, 100, and 200 mg/kg/d; after the initial tests, we finally selected 200 mg/kg/d for the formal experiment) [16,17,18]. DSS group mice were orally gavaged with 0.9% NaCl saline for 21 days. On the 15th day, 3% DSS (MP Biomedicals, Santa Ana, CA, USA) was added to the drinking water of the mice in the DSS group and the DSS-EPP group until the 21st day to establish a mouse model of acute colitis [19]. On the 14th day, fresh fecal particles were collected from the DSS group and the DSS-EPP group for follow-up experiments. The disease activity index (DAI) is a mature scoring system. The DAI score was observed and recorded on the 14th day. The DAI score reflects the combination of hematochezia, weight loss, and the physical morphology of the feces. The criteria were obtained from Ge et al. [20]. The experiment was terminated on the 21st day, and the mice were euthanized before colon tissues and blood samples were collected. The colitis and cecum contents were obtained, then stored in a −80 °C freezer for further investigation.

### 2.4. Fecal Microbial Transplantation (FMT)

Fresh fecal particles were collected from the DSS group and the DSS-EPP group (feces before the 15th day) as donors, sterile PBS (50 mg/mL) was added, and the mixture was homogenized. After homogenization, the fecal suspension was filtered with a 70 μm screen to remove large particles in the feces and then filtered with a 40 μm screen. The liquid obtained through the sieve was collected and resuspended in a sterile 50 mL centrifuge tube. Then, 10% sterile glycerol was added to the resuspended liquid; the centrifuge tube was sealed with sealing film and stored in a −80 °C freezer [21]. Seven-week-old BALB/C male mice were treated with a mixture of antibiotics [vancomycin (0.5 mg/mL), streptomycin (1 mg/mL), ampicillin (0.5 mg/mL), and gentamicin (1 mg/mL) acquired from Dalian Meilun Biotechnology Co. (Dalian, China)] in their drinking water for 14 days, after which the water was replaced every two days [22]. BALB/c male mice were divided into two groups. The F-DSS group received fecal suspensions from the DSS group, and the F-DSS-EPP group received fecal suspensions from the DSS-EPP group. Each animal received a fecal suspension of 200 mg/kg/d once every two days for a total of five treatments in 10 days as shown in Figure 1B. All processes were carried out under aseptic conditions.

### 2.5. Tissue Sections

The mouse colon tissue obtained was fixed using a 4% paraformaldehyde solution for an overnight period, followed by embedding in paraffin and cutting into slender sections of about 4 µm. The resultant slices underwent staining with hematoxylin and eosin (HE) in addition to Alcian blue (AB)–periodic acid–Schiff (PAS) [23]. After staining, histopathological features were observed, while after AB-PAS staining, pathological features, ranging from the loss of goblet cells to mucin reduction, were observed. We used the IHC-Toolbox plug-in in ImageJ to analyze the colon tissue-positive area after AB-PAS staining.

### 2.6. Immunofluorescence

Immunofluorescence was performed according to the relevant literature [24]. Colon sections were fixed, antigen-repaired, membrane-permeabilized, and blocked. Then, the colon tissue was incubated with ZO-1, occludin, and claudin-1 antibody overnight. On another day, the sections were stained with FITC-labeled secondary antibody for one hour. DAPI was used for nuclear staining; the sections were counterstained with DAPI for 10 min, followed by mounting and imaging.

### 2.7. RNA Extraction and RT-qPCR Analysis

Colon tissue RNA was extracted using an RNA extraction reagent (Ya Mei, Shanghai, China). The RNA was transcribed in reverse into cDNA using RTIII All-in-One Mix (Monad, Suzhou, China) according to the provided guidelines. Quantitative polymerase chain reaction (RT-qPCR) was then employed to assess the cDNA. With β-actin as the internal control, the relative expression level of the genes was determined by the 2^−ΔΔCT^ method. All the primer sequences used for RT-qPCR are shown in Table S1.

### 2.8. Western Blotting

Protein lysate buffer (RIPA) was added to the colon samples, and the RIPA buffer was adjusted to a concentration of 1 (PMSF):100 (RIPA) for protein extraction. Then, the colon was homogenized, and the sample was centrifuged at 14,000× *g* for 15 min to absorb the supernatant. Then, the protein buffer was added to the samples, after which they were boiled for 15 min at 95 °C. The samples were separated according to molecular weight by SDS-PAGE (10%) and then transferred to PVDF membranes. After the proteins were transferred to a membrane, the membrane was sealed for 30 min with rapid sealing solution (Yamei, Shanghai, China). After sealing, the appropriate primary antibody was added, and the sample was incubated overnight; following this, the membrane was washed with TBST, and the secondary antibody was added and combined with the primary antibody [23]. After visualizing the protein bands, we used ImageJ (V1.8.0) to perform grayscale analysis.

### 2.9. Enzyme-Linked Immunosorbent (ELISA) Assay

According to the operating instructions of an ELISA kit (SINOBESTBIO, Shanghai, China), we used the proportion of 1 g colon samples added with 9 mL PBS; a tissue grinder was used for homogenization. The blood samples and the prepared tissue homogenate were stored at 4 °C overnight. On the second day, the blood samples were centrifuged at 3000 rpm for 15 min. The supernatant was subsequently removed. LPS, IL-6, IL-1β, TNF-α, MDA, SOD, and T-AOC levels were determined via a mouse ELISA kit, after which the optical density (OD) was detected via an enzyme labeling instrument. Then, the concentration of each sample was calculated.

### 2.10. 16S rRNA Sequencing

Fecal samples were obtained, and a fecal DNA extraction kit (model D3141, Meiji Biotechnology Co., Ltd., Guangzhou, China) was used to extract fecal DNA. The purity of the DNA was determined by a NanoDrop microspectrophotometer (Model NanoDrop 2000, Semer Fischer Technology, Massachusetts, USA), and the integrity of the nucleic acid samples, whether they were degraded or whether the protein was contaminated, was determined by agarose gel electrophoresis. The extracted DNA served as a template for amplifying the V3–V4 region of 16S rDNA utilizing specific primers tagged with barcodes. The PCR products were purified by AMPure XP beads, and, after purification, the products were analyzed by using Quantitative Qubit 3.0. Next, the amplification products from the second row were purified by AMPure XP beads, quantified by an ABI StepOnePlus Real-Time PCR System (Life Technologies, Bedford, MA, USA), and sequenced on a computer according to the PE250 model of NovaSeq 6000. The Omismart tool at https://www.omicsmart.com/platform (accessed on 22 September 2023) was utilized for subsequent bioinformatics analysis.

### 2.11. LC-MS Nontargeted Metabolomic Analysis

The contents of the cecum were moved into Eppendorf tubes, with the collection of the appropriate sample material. A solution of methanol/acetonitrile/water (2:2:1, *v*/*v*) was added, pre-cooled, and the mixture was vortexed and ultrasonicated at −20 °C for 10 min. Subsequently, the mixture underwent centrifugation at 14,000× *g* for 15 min. The resulting supernatant was then subjected to vacuum drying, followed by the addition of 100 μL of aqueous acetonitrile (acetonitrile/water = 1:1, *v*/*v*). Centrifugation at 14,000× *g* for 15 min was conducted, with the supernatant collected for further examination. The analysis was carried out utilizing a UHPLC (1290 Infinity LC, Agilent Technologies, Santa Clara, CA, USA) in conjunction with a quadrupole time-of-flight (AB Sciex TripleTOF 6600) at Shanghai Applied Protein Technology Co., Ltd., Shanghai, China. The examination and assessment of nontargeted metabonomics were executed by Guangzhou Chideo Biotechnology Co., Ltd. (located in Guangzhou, China). The identification of differentially abundant metabolites was determined using Variable Importance in the Projection (VIP) values. The thresholds utilized to categorize the metabolites with differential abundance in the components were as follows: an expression of upregulation ≥ 1 or downregulation ≤ 1, a *p*-value of <0.05, and VIP ≥ 1.

### 2.12. Statistical Analyses

GraphPad Prism 9.5 (La Jolla, CA, USA) was used to perform statistical analyses. The data are expressed as the mean ± standard deviation (SD). A one-way analysis of variance (ANOVA) followed by Dunnett’s test were used to evaluate the differences among the groups. *p* values < 0.05 were considered to indicate statistical significance.

## 3. Results

### 3.1. EPP Ameliorates DSS-Induced Colitis in Mice

**Figure 2:**
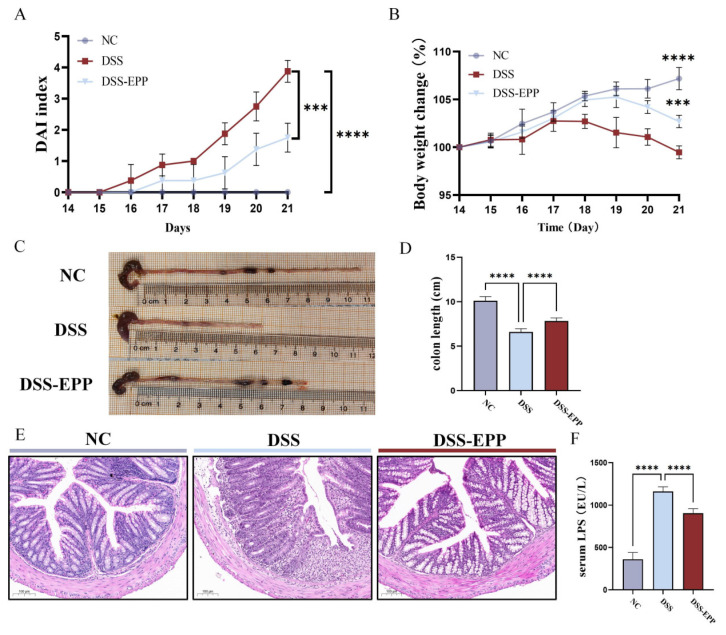
*Echinacea purpurea* polysaccharide (EPP) ameliorates DSS-induced colitis symptoms. (**A**) The DAI score during the course of colitis. (**B**) The weight change curve during the course of colitis. (**C**) Representative pictures of the colon. (**D**) The results of the statistical analysis of colon length in mice from each group were obtained. (**E**) The results of the HE staining of the pathological sections of colon tissue are shown. (**F**) The level of lipopolysaccharide (LPS) in the serum of mice was determined via ELISA kits. The data are presented as the mean ± SD (n = 8). *** *p* < 0.001 and **** *p* < 0.0001.

The methodology is illustrated in Figure 1A whereby mice from the DSS group and DSS-EPP group had 3% (*w*/*v*) DSS added to their drinking water on days 14–21. The DAI score of the colitis model mice were significantly higher (*p* < 0.001) and their body weights were significantly lower (*p* < 0.0001) compared to those of the NC group, and the length of the colon was significantly shorter (*p* < 0.0001). In contrast, EPP treatment significantly attenuated DSS-induced colitis symptoms (Figure 2A–D). H&E staining revealed inflammatory cell infiltration, intestinal epithelial cell loss, disordered arrangement, and mucosal swelling in the colonic tissue of the DSS treatment group. Treatment with EPP alleviated the above-described effects (Figure 2E). In addition, the serum LPS concentration increased significantly after DSS treatment; EPP treatment could reduce the serum LPS concentration (Figure 2F). The above results suggest that EPP can alleviate the clinical symptoms of DSS-induced colitis.

### 3.2. EPP Ameliorates DSS-Triggered Proinflammatory Responses by Inhibiting the TLR4-NF-κB Inflammatory Pathway

**Figure 3:**
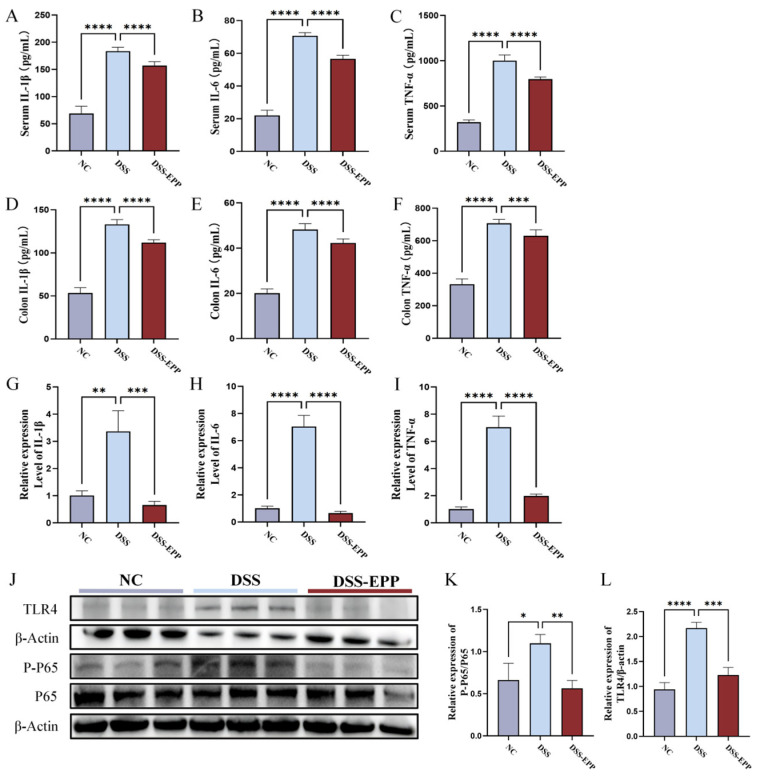
The effects of *Echinacea purpurea* polysaccharide (EPP) treatment on inflammation and inflammation-related pathways in mice. (**A**–**C**) The levels of inflammatory cytokines in the serum of mice in each group were measured via ELISA kits. (**D**–**F**) The levels of inflammatory cytokines in colon tissue were measured via ELISA kits. (**G**–**I**) The relative mRNA expression of inflammatory cytokines evaluated by RT-qPCR. (**J**) Representative Western blotting images of the TLR4, p65, p-p65, and β-actin proteins are shown. (**K**) The relative expression of p-p65/p65. (**L**) The relative expression of TLR4/β-actin. The data are presented as the mean ± SD, (n = 3–8). * *p* < 0.05, ** *p* < 0.01, *** *p* < 0.001 and **** *p* < 0.0001.

We evaluated the effects of EPP treatment on the DSS-induced proinflammatory response and oxidative damage. In comparison to those in the NC group, the serum and tissue levels of TNF-α, IL-β, and IL-6 in the DSS group were significantly increased (*p* < 0.0001). In the EPP group, this trend was significantly reversed (Figure 3A–F), and the change in the expression level of mRNA was consistent with these findings (Figure 3G–I). Western blotting revealed that the expression of TLR4 and the phosphorylation of the NF-κB protein in the DSS group were significantly increased compared to those in the NC group, and EPP pretreatment inhibited the activation of the TLR4-NF-κB pathway induced by DSS (Figure 3J–L). These results suggest that EPP intervention may alleviate colonic inflammation by inhibiting the TLR4-NF-κB pathway.

### 3.3. EPP Ameliorates DSS-Induced Oxidative Stress in Colon Tissue

**Figure 4:**
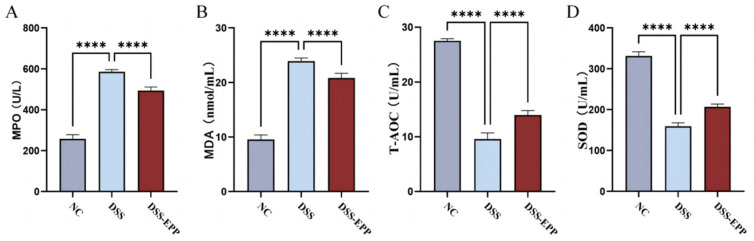
The effect of *Echinacea purpurea* polysaccharide (EPP) on oxidative damage. The levels of the oxidative stress index (**A**) MPO, (**B**) MDA, (**C**) T-AOC, and (**D**) SOD in colon homogenates were evaluated via ELISA kits. Data are presented as the mean ± SD (n = 8). **** *p* < 0.0001.

In comparison to the control group, the levels of MDA and MPO markedly increased in the DSS group (Figure 4A,B), with a significant decrease noted in SOD and T-AOC levels (Figure 4C,D). Notably, EPP therapy effectively prevented the decline in the tissue antioxidant index (*p* < 0.0001). In summary, EPP can alleviate DSS-induced oxidative stress in colon tissue.

### 3.4. EPP Alleviate DSS-Induced Intestinal Damage by Promoting the Tight Junction Protein

**Figure 5:**
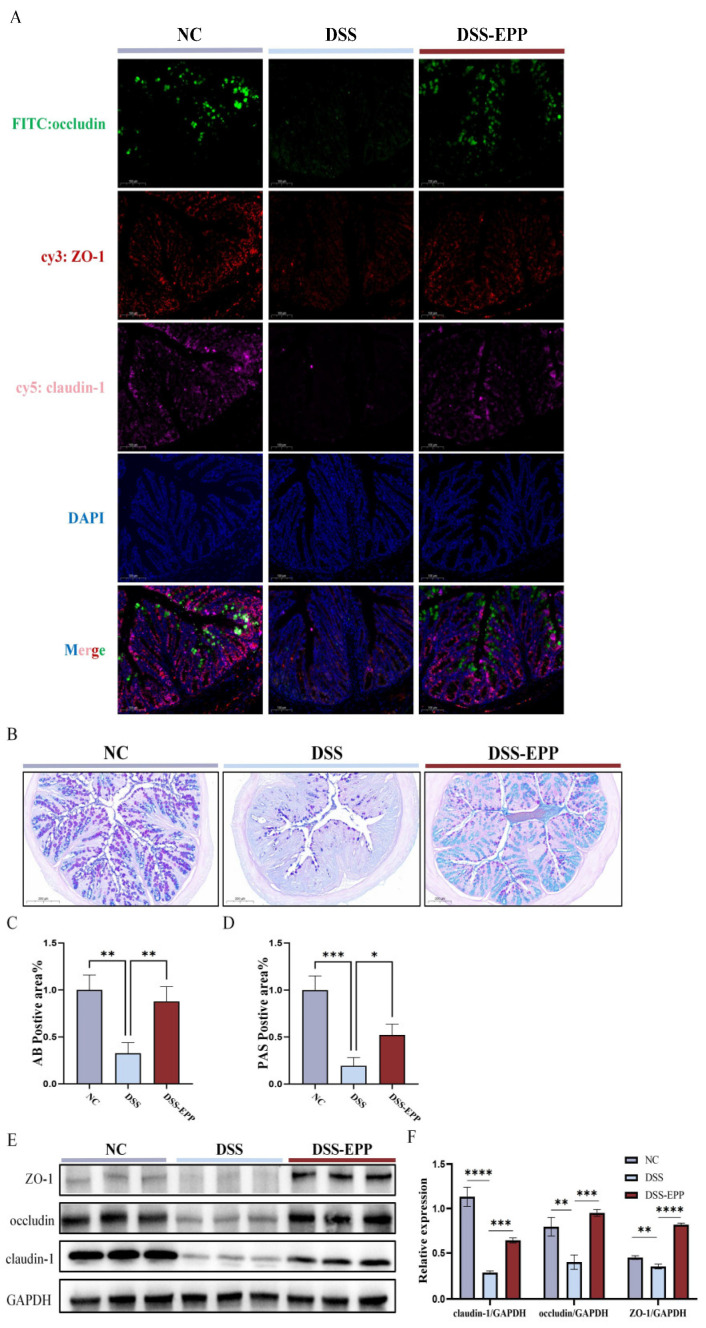
The recovery effect of EPP on intestinal barrier damage in DSS-induced mice. (**A**) Representative immunofluorescence images of ZO-1, occludin, and claudin-1 expression in colon sections (red fluorescence–cy3 represent ZO-1, green fluorescence–FITC represent occludin, pink fluorescence–cy5 represent claudin-1, and blue fluorescence–DAPI for nuclear) (**B**) Representative images of AB-PAS staining in the NC, DSS, and DSS-EPP groups (scale, 200 µm). (**C**) The results of the statistical analysis of AB-positive areas. (**D**) The results of the statistical analysis of PAS-positive areas. (**E**) The representative Western blotting images show ZO-1, occludin, and claudin-1 expression. (**F**) The relative expression of ZO-1/GAPDH, occludin/GAPDH and claudin-1/GAPDH are shown. Data are presented as the mean ± SD (n = 3). * *p* < 0.05, ** *p* < 0.01, *** *p* < 0.001, and **** *p* < 0.0001.

The intestinal epithelial barrier is crucial to the occurrence and development of UC. In patients with UC, the intestinal barrier is damaged, and intestinal epithelial cells fall off, which leads to an increase in the epithelial space [25]. Therefore, UC can be effectively relieved by improving intestinal epithelial barrier function. Thus, we used AB-PAS staining to evaluate the protective effect of EPP on the mucous layer of the colon. The AB-PAS staining results showed that DSS treatment led to the loss of goblet cells and decreased the secretion of glycoproteins (Figure 5B–D). EPP treatment promoted the recovery of glycoprotein and goblet cell density. Western blotting and immunofluorescence were used to evaluate the effect of EPP intervention on intestinal tight junction proteins (Figure 5A,E,F). The expression of claudin-1, occludin, and ZO-1 was significantly lower in the DSS group, while in the EPP intervention group, the expression of these three tight junction proteins was significantly increased (*p* < 0.001) (Figure 5E,F). These results indicated that EPP can alleviate DSS-induced intestinal damage by promoting the colon tight junction protein.

### 3.5. EPP Ameliorates DSS-Induced Intestinal Microbiota Disorder

**Figure 6:**
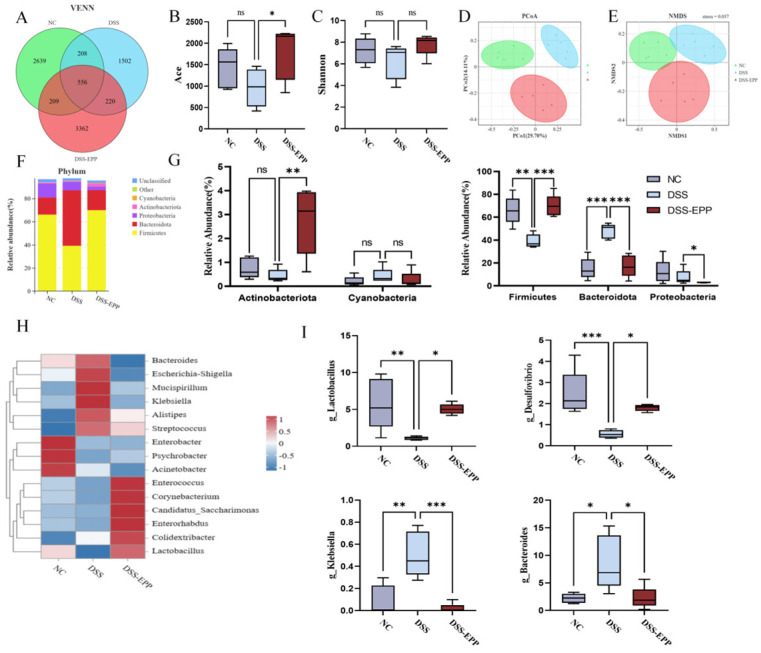
*Echinacea purpurea* polysaccharide (EPP) alleviated the disorder in the host intestinal microbiota. (**A**) The Venn diagram shows the OTUs that were detected in the NC, DSS, and DSS-EPP groups. (**B**,**C**) The Shannon and Ace indices indicate the α diversity. (**D**,**E**) The PCoA and NMDS methods were used to analyze the β diversity of the intestinal microbiota. (**F**) The composition of the intestinal microbiota at the phylum level was determined. (**G**) Differences in the abundance of the intestinal microbiota at the phylum level. (**H**) The heatmap shows the differentially abundant microbiota at the genus level. (**I**) The abundance of four intestinal microbiota (Bacteroides, Klebsiella, Lactobacillus, and Desulfovibrio). Data are presented as the mean ± SD (n = 5); ns, *p* > 0.05; * *p* < 0.05; ** *p* < 0.01, and *** *p* < 0.001.

Increasing evidence shows that disorders of the intestinal microbiota in the host are crucial to the occurrence and development of colitis [26]. Consequently, to examine the impact of EPP on the intestinal microbiota, we employed 16S rRNA sequencing. We found a total of 7503 OTUs in the samples. There were 2639 in the NC group, 3362 in the DSS-EPP group, and 1502 in the DSS group (Figure 6A). Alpha diversity was used to evaluate community richness and community diversity. Compared with those of the NC group, the Shannon and Ace indices of the DSS group exhibited a downward trend, while EPP alleviated the decrease in microflora (Figure 6B,C). Moreover, when evaluating the beta diversity of gut microorganisms, PCoA and NMDS analysis revealed that the intestinal microbial compositions of the three groups were significantly different (Figure 6D,E). The abundance of the Bacteroides in the DSS group was significantly increased compared with the NC group (*p* < 0.0001), while Firmicutes abundance decreased significantly (*p* < 0.01). However, EPP treatment significantly increased the abundance of Firmicutes and Actinobacteria (*p* < 0.01) and significantly decreased the abundance of Bacteroides and Proteobacteria (*p* < 0.05). Figure 6H shows the differences in microbiota at the genus level among the three groups in the form of a heatmap. The abundances of Bacteroides, Escherichia–Shigella, Mucispirillum, Klebsiella, Alistipes, and Streptococcus in the DSS group were significantly increased than those in the NC group and the EPP group (*p* < 0.05). Figure 6I shows the changes in the abundances of Bacteroides, Klebsiella, Lactobacillus, and Desulfovibrio, which are four intestinal microbiota, at the genus level. In summary, EPP can alleviate the UC-induced disruption of the intestinal microbiota.

### 3.6. EPP Ameliorates DSS-Induced Intestinal Metabolic Disorder

**Figure 7:**
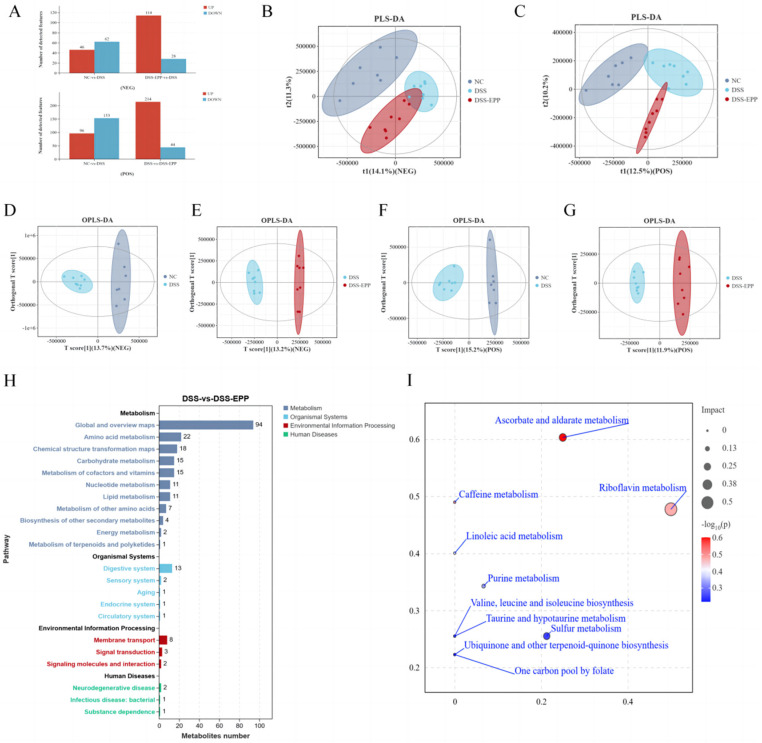
The effect of EPP on metabolic disorders. (**A**) Differentially abundant metabolites between NC and DSS, DSS and DSS-EPP were identified in two ionization modes (negative modes and positive modes). (**B**) The results of the PLS-DA analysis in each group in negative ion mode. (**C**) The results of the PLS-DA analysis in positive ion mode are shown. (**D**,**E**) The results of OPLS-DA between NC and DSS, DSS and DSS-EPP in anion mode, and (**F**,**G**) The OPLS-DA results in positive ion mode are shown. (**H**) The results of the KEGG enrichment analysis. (**I**) The results of the MetPA enrichment analysis are shown. (n = 7–8).

**Figure 8:**
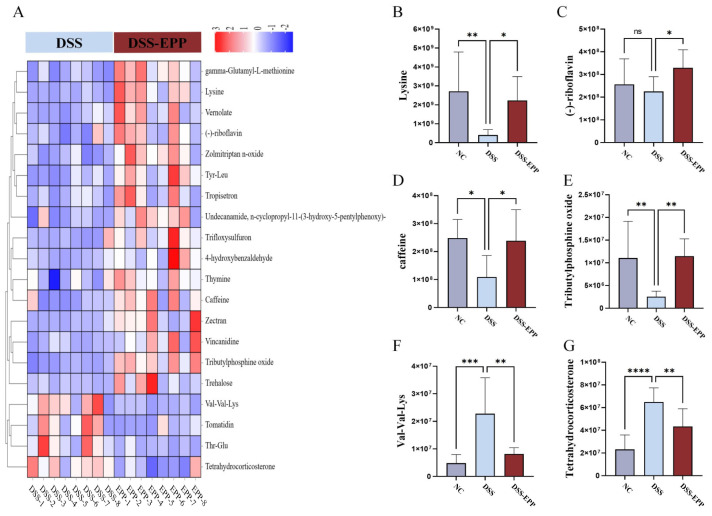
Differentially abundant metabolites. (**A**) The heatmap shows the top 20 metabolites with a fold change, and VIP value ≥ 1. (**B**–**G**) The abundances of 6 metabolites (lysine, (-)-riboflavin, caffeine, tributylphosphine oxide, Val-Val-Lys, and tetrahydrocorticosterone) in the NC, DSS, and DSS-EPP groups were determined. Data are presented as the mean ± SD (n = 7–8); ns, *p* > 0.05; * *p* < 0.05; ** *p* < 0.01; *** *p* < 0.001; and **** *p* < 0.0001.

In order to assess the impact of EPP on intestinal metabolism in DSS-induced colitic mice, we used LC-MS untargeted metabonomics to analyze the metabolic composition of cecal contents. In negative ion mode, we identified 62 metabolites with downregulated expression and 46 metabolites with upregulated expression in the DSS group compared with the NC group. Conversely, the DSS-EPP group displayed 114 metabolites with increased expression and 28 metabolites with decreased expression compared to the DSS group in the same mode; in positive ion mode, 153 metabolites had downregulated expression and 96 metabolites had upregulated expression in the DSS group compared with the control group. Compared with those in the DSS group, 214 metabolites had upregulated expression and 44 had downregulated expression in the DSS-EPP group (Figure 7A). PLS-DA and OPLS-DA revealed differences in metabolic characteristics among the three groups of samples (Figure 7B–G). Based on the KEGG database, a functional enrichment analysis of the differentially abundant metabolites was carried out, and the results showed that the differentially abundant metabolites were related to metabolism, the organismal system, and human diseases (Figure 7H). The results of the MetPA enrichment analysis showed that ascorbate and aldarate metabolism, caffeine metabolism, and riboflavin metabolism were the three metabolic pathways with the highest enrichment values (Figure 7I). Figure 8A shows the top 20 differentially abundant metabolites between the DSS group and the DSS-EPP group. Compared with those in the NC group, the abundances of lysine, (-)-riboflavin, caffeine, and tributylphosphine oxide in the DSS treatment group were significantly lower (*p* < 0.05), while EPP intervention significantly restored the abundances of these four metabolites (*p* < 0.05) (Figure 8B–E). Similarly, EPP treatment significantly inhibited the increase in Val-Val-Lys and Tetrahydrocorticosterone levels induced by DSS (Figure 8F,G). As detailed above, the results showed that EPP can alleviate intestinal metabolic disorder in DSS-induced mice.

### 3.7. Correlation Analysis of Differentially Abundant Intestinal Microbiota, Differentially Abundant Metabolites, Inflammation, and Oxidative Stress Indices

**Figure 9:**
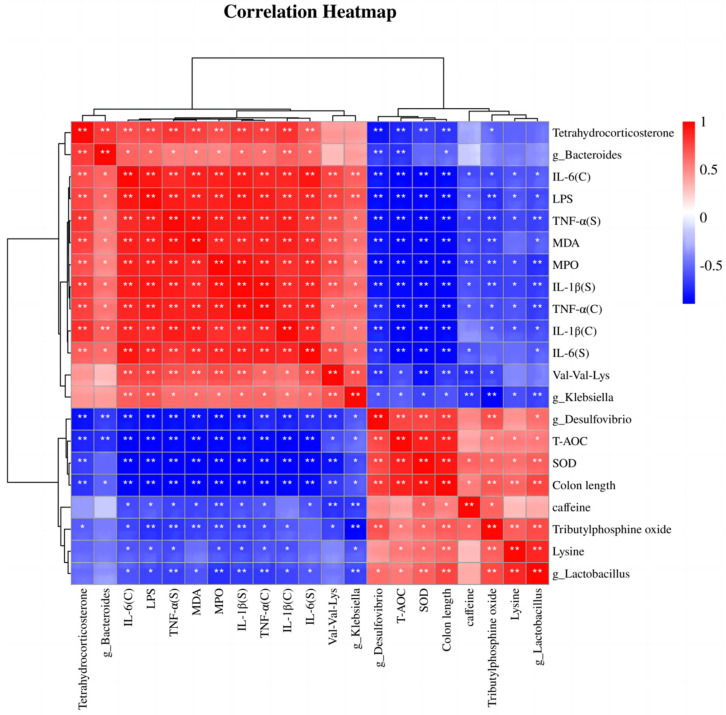
Spearman correlation analysis. “C” indicates colon homogenates, and “S” indicates serum. * *p* < 0.05 and ** *p* < 0.01.

We used Spearman correlation analysis to assess the correlations between four representative bacteria, three metabolites, and physiological and biochemical indices. As shown in Figure 9, Bacteroides and Klebsiella were positively correlated with MPO and MDA levels and with tissue and serum proinflammatory factors and negatively correlated with the expression of antioxidant factors, colon length, and two representative metabolites, lysine and caffeine. Lactobacillus and Desulfovibrio were positively correlated with antioxidant factors, lysine and caffeine metabolites, and colon length but negatively correlated with proinflammatory factors, pro-oxidative stress indices, and the two metabolites Val-Val-Lys and tetrahydrocorticosterone. The heatmap of the correlation analysis provides a basis for understanding the specific mechanism by which EPP alleviates colonic inflammation induced by DSS and reveals the positive and negative relationships among representative intestinal bacteria, metabolites, and the phenotypes of colonic inflammatory disease; these results provide a basis for future research.

### 3.8. Microbiota Transplantation from EPP-Treated Mice Ameliorates DSS-Induced Colitis

**Figure 10:**
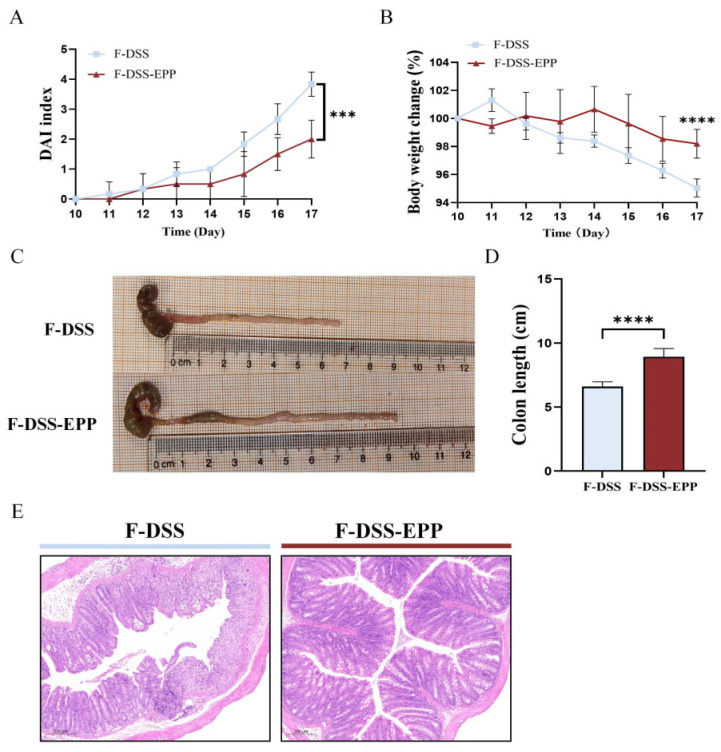
Protective effect of intestinal microbiota after EPP intervention on DSS-induced colitis. (**A**) DAI score during course of colitis. (**B**) Weight change curve during course of colitis. (**C**) Representative images of changes in colonic length of mice. (**D**) Statistical analysis of changes in colonic length in F-DSS and F-DSS-EPP groups. (**E**) H&E staining of F-DSS and F-DSS-EPP samples was performed. Data are presented as mean ± SD (n = 6); *** *p* < 0.001, and **** *p* < 0.0001.

To further assess the impact of the gut microbiota on UC relief with EPP, a fecal microbiota transplantation (FMT) experiment was carried out, the results of which are shown in Figure 1B. Compared with those in the F-DSS group, the F-DSS-EPP group had lower DAI scores (Figure 10A), less weight loss (Figure 10B), and significantly less colon shortening (Figure 10C,D) and diminished inflammatory cell infiltration, mucosal swelling, and intestinal epithelial cell loss (Figure 10E). In summary, these results suggest that the EPP alleviation of DSS-induced colitis can be achieved through FMT.

### 3.9. Microbiota Transplantation from EPP-Treated Mice Ameliorates DSS-Induced Inflammation and Oxidative Stress

**Figure 11:**
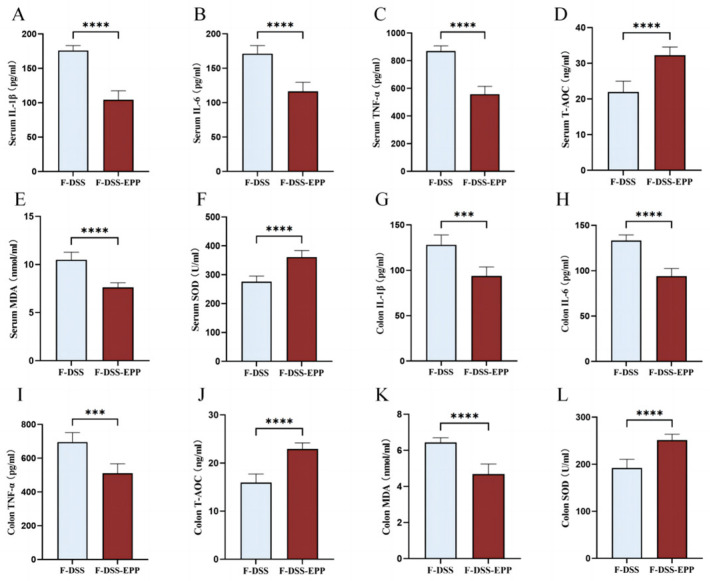
The effects of intestinal microbiota transplantation after EPP intervention on inflammation and oxidative stress. (**A**–**C**) The levels of IL-1β, IL-6 and TNF-α in serum were evaluated via ELISA kits. (**G**–**I**) The levels of IL-1β, IL-6 and TNF-α in colon tissue were determined. (**D**–**F**) The levels of oxidative stress indices T-AOC, MDA, SOD in serum. (**J**–**L**) The levels of T-AOC, MDA, SOD in colon tissue were determined via ELISA kits. *** *p* < 0.001, and **** *p* < 0.0001.

Serum and colonic tissue homogenate ELISA showed that the TNF-α, IL-1β, and IL-6 levels in the F-DSS-EPP group were significantly lower than those in the F-DSS group (Figure 11A–C,G–I), while the antioxidative indices T-AOC and SOD levels were significantly higher (*p* < 0.0001). The level of MDA was significantly lower (*p* < 0.0001) (Figure 11E,K). In summary, these results suggest that the EPP inhibition of inflammation and oxidative stress can be mediated through FMT.

## 4. Discussion

Aminosalicylic acid formulations, like mesalazine, are frequently utilized in clinical practice as initial pharmacological options for managing mild-to-moderate UC symptoms, and higher doses of mesalazine are more effective for severe conditions; however, the potential for dose-related toxicity poses a significant challenge for patients. Therefore, there is an urgent need to explore new treatments with better efficacy and lower dose-dependent toxicity for patients with UC.

*Echinacea purpurea* is cultivated as a plant medicine in Europe and North America, mainly for the prevention and mitigation of diseases related to upper respiratory tract infection. According to recent research, EPP can prevent alcohol-induced liver injury by protecting the intestinal barrier and regulating inflammation [18]. EPP can also regulate changes in microbiota and metabolites [27]. However, the effects of *Echinacea purpurea* polysaccharides on inflammation, oxidative damage, intestinal barrier damage, intestinal microbiota disorders, and metabolite changes caused by UC are still unclear. Our results showed that EPP treatment can alleviate DSS-induced colitis symptoms, such as weight loss, an increased DAI score, colon shortening, and histopathological features. These results provide a basis for dietary supplementation with EPP to alleviate UC.

In the occurrence and development of UC, the intestinal mechanical barrier is damaged, and bacteria or harmful metabolites enter the blood to stimulate the immune system, resulting in the release of the pro-inflammatory factors IL-1β, IL-6, and TNF-α, which promote the inflammatory response [28,29]. TLR4 is the specific receptor for LPS [30]. After binding to a ligand, NF-κB phosphorylation can occur through signal transduction, after which the levels of IL-1β, IL-6, TNF-α, and other inflammatory factors are regulated to promote the inflammatory response [30,31]. In accordance with these findings, our results showed that after DSS treatment, the intestinal barrier was destroyed, and the serum LPS concentration increased significantly. After EPP treatment, the level of LPS decreased, and EPP inhibited the TLR4/NF-κB inflammatory pathway. Therefore, EPP may alleviate intestinal inflammation by inhibiting the TLR4/NF-κB inflammatory pathway.

The intestinal microbiota plays a very important role in the intestinal physiological process and immunity in the host [11]. Increasing evidence shows that the abundance of Firmicutes decreases and that the abundances of Bacteroides and Proteobacteria increase in mice with UC [32]. Changes in these flora also represent an imbalance in the intestinal microbiota in the host [33]. Consistent with recent studies, our results showed that DSS treatment greatly reduced the abundance of Firmicutes while increasing the abundance of Bacteroides. However, EPP intervention significantly increased the abundance of Firmicutes and decreased the abundance of Bacteroides and Proteobacteria. At the genus level, DSS treatment significantly increased the abundance of Escherichia–Shigella, Klebsiella, and Alistipes [34,35,36], while the abundance of these three harmful bacteria was decreased after EPP treatment. Interestingly, although there was no significant difference between the control group and the model group, the levels of Candidatus_Saccharimonas, Enterorhabdus, and Corynebacterium increased significantly after EPP treatment. Our study revealed that DSS treatment decreased Lactobacillus levels, while EPP treatment significantly increased Lactobacillus levels. Lactobacillus species are recognized as beneficial bacteria in IBD and play a certain role in the immune barrier, intestinal epithelial barrier, and mucus barrier of patients. Studies have shown that *Lactobacillus reuteri* GroEL can inhibit macrophage M1 polarization, promote M2 polarization, and inhibit the inflammatory response in the body [37]. Many kinds of lactic acid bacteria, such as *Lactobacillus acidophilus* KLDS 1.0901, *Lactobacillus plantarum* KLDS 1.0318, and *Lactobacillus helveticus* KLDS 1.8701, can alleviate damage to the intestinal epithelial barrier [38]. Tight junctions (TJs) widely exist on the outer surface of intestinal epithelial cells [39]. As an important component, the intestine seals the space between the intestinal epithelium, maintains cell polarity, and helps maintain intestinal permeability [40]. TJs can be harmed by DSS treatment, which increases the intestinal permeability and promotes bacterial translocation [41,42,43]. According to our findings, EPP intervention could significantly promote the ZO-1, occludin, and claudin-1 tight junction protein. AB-PAS staining also confirmed the effect of EPP on the mucous layer, and EPP repaired the intestinal mechanical barrier in UC mice. In summary, EPP alleviates DSS-induced colitis in mice by repairing intestinal barriers (the mechanical barrier and the intestinal microbiota barrier) and regulating the intestinal microbiota balance.

In the process of intestinal digestion, the intestinal microbiota produces many metabolites that can interact with the intestinal epithelium and enter the host circulatory system. Changes in the intestinal flora can also cause changes in intestinal metabolites, which are significantly related to health or disease maintenance [44]. To explore the effect of EPP on intestinal metabolites, we carried out an LC-MS untargeted metabolomic analysis. The results showed that EPP treatment could restore the upregulated or downregulated expression of lysine, caffeine, and other metabolites in DSS-induced mice. The abundance of the metabolite lysine has been reported to decrease significantly in DSS-treated model mice [45,46]. Caffeine is a xanthine alkaloid [47], and caffeine intake also regulates redox reactions and inflammation. Caffeine intake can reduce ROS and lipid peroxidation and increase tissue glutathione (GSH) levels [48]. In our study, intervention with EPP restored the abundance of lysine and caffeine just as expected. Combining with pathway enrichment analysis, we found that DSS treatment decreased the abundance of lysine, (-)-riboflavin, and caffeine, while EPP treatment restored the abundance of these metabolites, indicating that EPP can reshape metabolism and play an anti-UC role.

At present, FMT has become a widespread research hotspot for the treatment of IBD [49,50]. Reshaping the intestinal microbiological ecosystem is also recognized as an effective strategy [51]. In this study, we used FMT to further demonstrate that intestinal microbiota transplantation after EPP remodeling can alleviate the symptoms of UC in mice.

However, our study has several limitations. Firstly, our study showed a notable enhancement in DSS-induced UC with EPP, confirmed through multidimensional analysis; the optimal dosage and potential adverse effects of EPP in human participants still need to be determined. Secondly, this study did not directly explore the changes in key intestinal bacteria or metabolites in their respective regulatory effects during the treatment of UC with EPP. Thirdly, our study suggests that the transplantation of the gut microbiota after EPP intervention can exert anti-UC effects, while it remains unknown whether the effects of EPP are exerted exclusively through the gut microbiota; these findings should be used in a follow-up in-depth study.

## 5. Conclusions

Overall, our studies have shown that EPP can ameliorate colitis clinical symptoms; repair the mechanical barrier by promoting the tight junction protein; reshape the microbial intestinal barrier by reducing the abundance of Bacteroides, Escherichia–Shigella, and Klebsiella, increasing the abundance of Lactobacillus; and regulate and reshape metabolism in UC mice, thereby reducing LPS entry into the blood and inhibiting LPS-induced TLR4-NF-κB signal transduction. In summary, our research showed that EPP can be used as a daily dietary supplement to prevent and alleviate UC and that EPP is promising for use as a mild drug for the treatment of UC.

## Data Availability

The data presented in this study are available on request from the corresponding author. The data are not publicly available due to privacy.

## Supplementary Materials

The following supporting information can be downloaded at https://www.mdpi.com/article/10.3390/nu16091305/s1, Table S1: All primers.

## Abbreviations

UC, ulcerative colitis; EPP, *Echinacea purpurea* polysaccharide; IBD, inflammatory bowel disease; DSS, dextran sulfate sodium; TLR4, Toll-like receptor 4; NF-κB, nuclear factor kappa-B; 5-ASA, 5-aminosalicylate; LPS, lipopolysaccharide; TNF-α, tumor necrosis factor-α; IL, interleukin; Nrf2, NF-E2-related factor; ZO-1, zonula occludens 1; NC, normal control; DAI, disease activity index; FMT, fecal microbial transplantation; PLS-DA, partial least squares discriminant analysis; ELISA, enzyme-linked immunosorbent assay; OPLS-DA, orthogonal partial least squares discriminant analysis; VIP, variable importance prediction; SD, standard deviation; ANOVA, one-way analysis of variance; HE, hematoxylin–eosin; AB-PAS, Alcian blue–periodic acid–Schiff; PCoA, principal coordinates analysis; NMDS, Non-metric multidimensional scaling; TJ, tight junction.
